# Examining the Medical Blogosphere: An Online Survey of Medical Bloggers

**DOI:** 10.2196/jmir.1118

**Published:** 2008-09-23

**Authors:** Ivor Kovic, Ileana Lulic, Gordana Brumini

**Affiliations:** ^2^Department of Medical InformaticsRijeka University School of MedicineCroatia; ^1^Rijeka University School of MedicineCroatia

**Keywords:** Blog, journalism, medical, authorship, Internet, questionnaires, electronic publishing, medicine, podcast

## Abstract

**Background:**

Blogs are the major contributors to the large increase of new websites created each year. Most blogs allow readers to leave comments and, in this way, generate both conversation and encourage collaboration. Despite their popularity, however, little is known about blogs or their creators.

**Objectives:**

To contribute to a better understanding of the medical blogosphere by investigating the characteristics of medical bloggers and their blogs, including bloggers’ Internet and blogging habits, their motivations for blogging, and whether or not they follow practices associated with journalism.

**Methods:**

We approached 197 medical bloggers of English-language medical blogs which provided direct contact information, with posts published within the past month. The survey included 37 items designed to evaluate data about Internet and blogging habits, blog characteristics, blogging motivations, and, finally, the demographic data of bloggers.

Pearson’s Chi-Square test was used to assess the significance of an association between 2 categorical variables. Spearman’s rank correlation coefficient was utilized to reveal the relationship between participants’ ages, as well as the number of maintained blogs, and their motivation for blogging. The Mann-Whitney U test was employed to reveal relationships between practices associated with journalism and participants’ characteristics like gender and pseudonym use.

**Results:**

A total of 80 (42%) of 197 eligible participants responded. The majority of responding bloggers were white (75%), highly educated (71% with a Masters degree or doctorate), male (59%), residents of the United States (72%), between the ages of 30 and 49 (58%), and working in the healthcare industry (67%). Most of them were experienced bloggers, with 23% (18/80) blogging for 4 or more years, 38% (30/80) for 2 or 3 years, 32% (26/80) for about a year, and only 7% (6/80) for 6 months or less. Those who received attention from the news media numbered 66% (53/80). When it comes to best practices associated with journalism, the participants most frequently reported including links to original source of material and spending extra time verifying facts, while rarely seeking permission to post copyrighted material. Bloggers who have published a scientific paper were more likely to quote other people or media than those who have never published such a paper (*U*= 506.5, *n_1_*= 41, *n_2_*= 35, *P*= .016). Those blogging under their real name more often included links to original sources than those writing under a pseudonym (*U*= 446.5, *n_1_*= 58, *n_2_*= 19, *P*= .01). Major motivations for blogging were sharing practical knowledge or skills with others, influencing the way others think, and expressing oneself creatively.

**Conclusions:**

Medical bloggers are highly educated and devoted blog writers, faithful to their sources and readers. Sharing practical knowledge and skills, as well as influencing the way other people think, were major motivations for blogging among our medical bloggers. Medical blogs are frequently picked up by mainstream media; thus, blogs are an important vehicle to influence medical and health policy.

## Introduction

A blog (a portmanteau of Web log) is a website where entries are commonly displayed in reverse chronological order [1]. The history of blogs is closely connected with the history of the Internet itself. One of the world’s first websites was started in 1992 by Tim Berners-Lee, the inventor of the World Wide Web [2], and featured a news section pointing to new websites as they came online [3]. This website can be considered the earliest predecessor of modern blogs. Most of the people who were creating websites at that time were computer experts using them as their online diaries to write about their personal lives. The actual term “Web log” was coined in 1997 and shortened to “blog” in 1999 [1]. It was not until 1999 that blogging started to become popular among a broader population of Internet users, coinciding with the launch of LiveJournal and Blogger [4,5], the first dedicated blog hosting services. These blog publishing systems allowed individuals to create and maintain their own blogs without knowing hypertext markup language (HTML). In fact today, due to the constant advancement of such blog hosting sites, people with basic computer skills can start publishing their own blogs for free in a matter of minutes.

According to report by the Pew Internet and American Life Project (Pew), published in July of 2006, 8% (12 million) of 147 million adult users of the Internet in the United States keep a blog, while 39% (57 million) read one [6]. This represents a significant increase in numbers of both writers and readers of blogs from data published a year before by the same organization [7]. Additional evidence of the persistent growth of the “blogosphere”, a collective term encompassing all blogs and their interconnections, comes from the December 2007 issue of the Web Server Survey by Netcraft [8], an Internet monitoring company. This widely respected survey, that attempts to contact each and every website that is accessible on the Internet, credited blogs as the major contributor to the increase of nearly 50 million new websites in 2007 [9]. Technorati [10], the largest Internet search engine for blogs, claims to be currently tracking 112.8 million blogs, with 175,000 new blogs emerging every day.

Modern blogs have come a long way from their ancestors, evolving both technically and in terms of content. They can combine text, images, audio and video content, and links to other blogs, web pages, and other media related to their topic. Most blogs encourage feedback by allowing readers to leave comments, and it is through this knowledge sharing and debating process that they often engage a large and loyal readership. Many bloggers (blog authors) have ventured into professional journalism and have made a career out of writing blogs [11,12]. Their blogs are highly influential and are considered to be invaluable to readers who wish to follow the progress of a certain topic.

Despite their popularity, the effects of blogs, and among them medical blogs, are still largely unclear. However, it is well known that many debates originating among top medical bloggers have ended up in the pages of respectable journals, such as The New York Times, or even in news sections of the core scientific journals [13,14].

Scientific studies of medical blogs and their creators have so far been very scarce, although there is a great need for such research to help in the better utilization of blogs for the enhancement of teaching and learning productivity, advancements in scientific research, and support for continuing medical and patient education [15].

The goal of our study was to contribute to a better understanding of the medical blogosphere by investigating the characteristics of medical bloggers and their blogs. We sought to gain a better understanding of who these new medical storytellers are, what they write about, when, where, and how they do it, and finally what their motives for blogging are.

## Methods

### Survey

We used an online survey aimed at medical bloggers. In order to identify potential participants, we consulted 4 different websites, which aggregate medical blogs for their readers (Table 1).

We visited and took WebCite snapshots of these websites on February 10, 2007. The total number of blogs found on these websites was 740, but cross-referencing between the sources revealed 113 identical blogs, leaving us with 627 unique ones. All of these blogs were examined independently by all authors and checked against inclusion criteria, which were English-language medical blogs providing direct contact to the author, either via e-mail or online contact form, with posts published within the past month.

A medical blog was defined as a blog whose main topic was related to health or medicine. Out of the 627 listed blogs, 126 no longer existed, 10 turned out not to be medical blogs, 8 were written in languages other then English, and 152 were inactive, since their last posts were more than one month old. Eventually, a first examination of the blogs revealed 331 (53%) active and English-language medical blogs, out of which 143 (36%) were published on personal websites and 188 (64%) were published on blog hosting services. All of these blogs were once again visited and thoroughly inspected for the authors’ contact information. Contact information was found on 197 (60%) of these 331 blogs (169 e-mail addresses and 28 online contact forms), which in 103 (52%) cases were hosted on personal websites and in 94 (48%) cases on blog hosting services.

**Table 1:** Characteristics of the websites used to identify medical bloggers and data about number of indexed blogs on February 10, 2007

Name	Web address	Description	Blog enlisting procedure	Number of indexed blogs
Medgadget	http://www.medgadget.com/archives/2006/12/the_2006_medical_blog_nominees.html	Independent on-line journal covering the latest medical gadgets and technologies, discoveries in medical science, and the progress of the digital revolution in healthcare industries.	Blogs are nominated for the prestigious annual Medical Weblog Awards by the readers and verified by the editors.	118
Medlogs	http://www.medlogs.com	News aggregator for medical topics offering one of the most comprehensive listings of medical blogs available.	Readers send requests for inclusion of their blogs, which are later evaluated by the editors.	383
Trusted.MD Network	http://trusted.md/bloggers	The world’s largest community of health and medical bloggers acting as a conduit for connecting bloggers with their audience and institutions.	Only registered users can add their blogs to the listing.	173
Yahoo! Directory	http://dir.yahoo.com/Health/News_and_Media/Blogs/	Human created and maintained library of websites organized into categories and subcategories.	Readers suggest a blog for a Health/News and Media/Blogs subcategory, which is then reviewed by the editors.	66

Invitations to participate in the survey were sent in 3 phases to these 197 bloggers. While 190 invitations were delivered, 5 e-mail addresses were not working and 2 online contact forms provided error messages when we tried to submit the invitation. The overall response rate was 42% (80), with 52 (65%) bloggers completing the survey after the first invitation on February 20th, 17 (21%) after the second invitation on March 7th, and 11 (14%) after the third invitation on March 22, 2007. The invitation guaranteed anonymity and confidentiality to the respondents and also informed them that the gathered data would be published in a scientific journal.

Design of the survey questions was influenced by the Pew’s Blogger Callback Survey [6], with crucial modifications made to better address specific issues regarding medical bloggers and their blogs. The survey consisted of 37 questions divided into 4 parts: A – data about Internet and blogging habits (7 questions), B – data about blog characteristics (19 questions), C – data about blogging motivation (3 questions), and D – demographic data (8 questions) (see Multimedia Appendix 1 for the complete instrument).

### Statistical Analyses

Pearson’s Chi-Square test was used to assess the significance of an association between 2 categorical variables, for example gender and various blogging habits. Spearman’s rank correlation coefficient (rho), was utilized to measure the strength of a relationship between 2 ordinal-level variables. It was used to reveal the relationship between participants’ ages, as well as the number of maintained blogs, and their motivation for blogging. Finally, the Mann-Whitney U test was employed for measuring association significance of dependent variables at ordinal level and dichotomous independent variables. It was performed to reveal relationships between practices associated with journalism and participants’ characteristics such as gender and pseudonym use. All statistical values were considered significant when the *P* value was less than 0.05. Statistical analysis of data was performed using Statistica for Windows, release 7.1 (Statsoft, Inc., Tulsa, OK, USA).

## Results

### Demographic Characteristics and Internet Habits

The majority of the surveyed bloggers were white, highly educated males, between the ages of 30 and 49, working in healthcare industry, and residing in the United States (Table 2).

**Table 2:** General demographic characteristics of the responding medical bloggers (N=80)

Variable	n (%)
**Age**	
18-29	15 (19)
30-49	46 (58)
50-64	15 (19)
≥65	1 (1)
Missing	2 (3)
**Gender**	
Male	47 (59)
Female	25 (32)
Missing	7 (9)
**Education**	
Graduated high school	1 (1)
Some college	1 (1)
Graduated college	12 (16)
Some postgraduate college	8 (10)
Masters degree or doctorate	56 (71)
Missing	1 (1)
**Race**	
Asian	9 (11)
Black	1 (1)
White	59 (75)
Other	3 (4)
Missing	7 (9)
**Country of residence**	
Australia	4 (5)
India	2 (3)
Philippines	2 (3)
United Kingdom	5 (6)
United States of America	57 (72)
Other	8 (10)
Missing	1 (1)
**Job title**	
Faculty staff	4 (5)
Manager	14 (18)
Medical student	5 (6)
Nurse	5 (6)
Other medical profession	7 (9)
Physician	27 (34)
Researcher	4 (5)
Writer	4 (5)
Other	4 (5)
Missing	5 (7)
**Industry**	
Education	7 (9)
Healthcare	53 (67)
Information	2 (3)
Media	5 (6)
Pharmaceutical	2 (3)
Other	5 (6)
Missing	5 (6)

The majority of respondents, 56% (45/80), were heavy Internet users, spending 20 or more hours per week on the Internet, with almost all (77/80, 96%) utilizing a broadband Internet connection. About 99% (79/80) of the respondents reported that they get medical news from the Internet, 86% (69/80) from other blogs, 75% (60/80) from e-mail newsletters, 63% (50/80) from Really Simple Syndication (RSS) feeds, and 24% (19/80) from podcasts. Concerning traditional news sources, 72% (58/80) of the bloggers reported that they read newspapers, 56% (37/80) read magazines, 53% (34/80) watch television, and 39% (31/80) listen to the radio to access medical news. Male bloggers used RSS feeds to get medical news more than their female colleagues (79% vs 52%, *χ^2^_1_*= 4.86, *P*= .03). Mass media and blogs together were a preferred source of medical news for 54 (70%) of the respondents, while 15 (19%) expressed a preference for mass media only and 9 (11%) only for blogs.

### Blogging habits

Almost half (38/80, 47%) of the surveyed bloggers had a personal website before launching their blog. The majority of the participants can be considered experienced medical bloggers, since 23% (18/80) of them were blogging for 4 or more years, 38% (30/80) for 2 or 3 years, 32% (26/80) for about a year, and only 7% (6/80) for 6 months or less. Bloggers mainly wrote under their real name (60/80, 75%), as opposed to using a pseudonym (20/80, 25%). They preferred to write at home (64/80, 80%), rather than at work (16/80, 20%). For 35 (42%) of the participants, blog writing occupied 1 to 5 hours a week; another 22 (27%) reported that they invest 6 to 9 hours a week; and 15 (19%) bloggers reported that they write for more than 10 hours a week, while 10 (12%) of them do so for less than 1 hour a week. Most respondents had 1 blog (44/80, 55%), but there were those with 2 (17/80, 21%), and 3 or more blogs (19/80, 24%). In their main blog, more bloggers wrote about several different topics (63/80, 79%) as opposed to about only one topic (17/80, 21%) (Table 3).

Regarding the publishing of multimedia material on blogs, 63 (79%) bloggers reported that they posted photos, 60 (75%) posted images other then photos, 25 (31%) posted video files, and 26 (23%) posted audio files. RSS feeds where offered to readers by 89% (71) and e-mail newsletters by 40% (32) of blog writers. Those who got medical news from RSS feeds where more likely to offer RSS feeds to their readers (*χ^2^_1_*= 9.00, *P*= .003). Again, as in the case of receiving medical news via RSS feeds, female bloggers were less likely to offer RSS feeds to their readers than male bloggers (72% vs 98%, *χ^2^_1_*= 7.39, *P*= .006).

**Table 3:** Representation of main blog topics among the responding medical bloggers (N=80)

Main topic	n (%)
Health economics and policies	5 (6)
Information technology in medicine	10 (12)
Medical education	6 (8)
Medicine in general	6 (8)
Patient experience	4 (5)
Personal life	3 (4)
Pharmaceutical industry	4 (5)
Public health	9 (11)
Specific illness	8 (10)
Specific medical specialty	18 (23)
Missing	6 (8)

### Journalistic activities

More than half of the responding medical bloggers have published a scientific paper (43/80, 54%), 35 (44%) bloggers have published a book or a chapter in a book, and 32 (41%) have published a newspaper article. Highly educated bloggers were more likely to have published a book or a chapter in a book (50% vs 14%, *χ^2^_1_*= 6.19, *P*= .01) and a scientific paper (62% vs 21%, *χ^2^_1_*= 7.57, *P*= .08) than those with lower levels of education. When it comes to best practices associated with journalism, the participants most frequently reported including links to original source of material and spending extra time verifying facts, while they rarely tried to obtain permission to post copyrighted material (Table 4).

**Table 4:** Representation of practices associated with journalism among the responding medical bloggers (N=80)

Practice	Often n (%)	Sometimes n (%)	Hardly ever n (%)	Never n (%)	Doesn’t apply n (%)
Include links to original sources	73 (91)	6 (8)	0 (0)	0 (0)	1 (1)
Spend extra time verifying facts	47 (59)	27 (34)	4 (5)	1 (1)	1 (1)
Quote other people/media directly	42 (52)	25 (31)	10 (13)	2 (3)	1 (1)
Post corrections	23 (29)	37 (46)	0 (0)	18 (22)	2 (3)
Try to obtain permission for copyrighted material	6 (7)	17 (21)	21 (27)	28 (35)	8 (10)

Female medical bloggers were found to get permission for posting copyrighted material more often than male bloggers (*U*= 386, *n_1_*= 25, *n_2_*= 44, *P*= .03). Bloggers who have published a scientific paper were more likely to quote directly other people or media than those who never published such a paper (*U*= 506.5, *n_1_*= 41, *n_2_*= 35, *P*= .016). Blog writers who were blogging under their real name were more inclined to include links to original sources than those writing under a pseudonym (*U*= 446.5, *n_1_*= 58, *n_2_*= 19, *P*= .01).

### Motivations for Blogging

Major motivations for blogging were sharing practical knowledge or skills with others, influencing the way other people think, and expressing oneself creatively. Making money and staying in touch with friends and family were not reasons to blog for a majority of the participants (Table 5).

**Table 5:** Motivations for blogging of the responding medical bloggers (N=80)

Motivations for blogging	Major reason n (%)	Minor reason n (%)	Not a reason n (%)
To share practical knowledge and skills	59 (74)	19 (23)	2 (3)
To influence the way other people think	45 (56)	29 (36)	6 (8)
To express creatively	42 (53)	29 (36)	9 (11)
To document personal experiences	40 (50)	21 (26)	19 (24)
To motivate others to action	38 (48)	29 (36)	13 (16)
To store resources of information	34 (42)	24 (30)	22 (28)
To entertain people	22 (28)	32 (40)	26 (32)
To improve writing skills	21 (26)	34 (43)	25 (31)
To network or meet new people	15 (19)	33 (41)	32 (40)
To stay in touch with friends and family	9 (11)	10 (13)	61 (76)
To make money	6 (8)	23 (28)	51 (64)

Younger bloggers were more likely than their older peers to blog to improve their writing skills (*r*= .33, *P*= .049) and to write for the reason of entertaining their readers (*r*= .32, *P*= .008).

To bloggers who had more than one blog, staying in touch with friends and family was an important reason for blogging, as opposed to those with one blog (*r*= .31, *P*= .09).

### Blog Attention

Almost all of the respondents received attention for their blogs from other bloggers (79/80, 99%), 78% (62/80) from their coworkers or colleagues, 66% (53/80) from news media, and 47% (38/80) from their families. Receiving family attention was more common for those who had more than one blog (63% vs 33%, *χ^2^_2_*_=_ 7.13, *P*= .028). Bloggers who wrote using their real name rather than a pseudonym received more attention from their coworkers or colleagues (86% vs 58%, *χ^2^_1_*_=_ 6.97, *P*= .008), as did those who got medical news from other blogs (82% vs 43%, *χ^2^_1_*_=_ 5.89, *P*= .02). Three factors proved to be important in getting news media attention: more years of experience as a blogger (77% vs 48%, *χ^2^_3_*_=_ 10.23, *P*= .02), more hours per week spent on the Internet (70% vs 30%, *χ^2^_3_*_=_ 8.29, *P*= .04), and acquiring medical news from other blogs (71% vs 29%, *χ^2^_1_*_=_ 8.72, *P*= .01).

## Discussion

### Principal Findings

Our study has shed some light on the medical blogosphere by examining the characteristics of medical bloggers and their blogs. Responding medical bloggers were highly educated and devoted blog writers, faithful to their sources and readers. Such conduct has assured them attention from other bloggers, their coworkers and colleagues, as well as the news media.

Design of our survey questions was strongly influenced by the Pew’s Blogger Callback Survey [6], a well-constructed survey, which, with minor modifications, perfectly matched our needs. Furthermore, utilizing it allowed an opportunity for comparison of our bloggers with those from the Pew’s survey [6]. This survey was conducted through telephone interviews between July 5, 2005 and February 17, 2006, producing a sample of 233 general bloggers from the United States. There were numerous differences between our participants and those of the Pew’s survey [6]. Most of our bloggers were male, between 30 and 49 years old, with a high percentage of those holding a master’s degree or a doctorate. By contrast, the majority of Pew bloggers were younger, 19-29 years of age, evenly split between genders, and less educated. These bloggers were mainly writing about their personal lives (personal diaries or journals), with only 1% of them reporting health (general health, an illness) as the main subject of their blogs [6]. Similar findings have been reported by McKenzie, who surveyed 127 writers of personal journal blogs from the United States [16]. Her respondents were also younger and less educated than our sample, but with a strong prevalence of female bloggers [16]. It might be the case that writing about demanding topics like medicine, requires a certain age, as well as a higher degree of education and experience. The fact that 75% of our medical bloggers wrote under their real name, as opposed to only 45% of Pew bloggers [6], further emphasizes their maturity.

When it comes to medical news sources, our bloggers demonstrated themselves to be avid consumers of online sources as well as traditional ones, like newspapers and magazines. Among other technically advanced social media, only podcasts were used for receiving medical news by a small percentage of medical bloggers. They are a relatively new development, so it might be that medical bloggers are still not sufficiently informed about their many benefits [15,17,18].

Responding medical bloggers demonstrated a captivating level of adherence to best practices generally associated with journalism. All of them included direct contact information on their blogs. They also included links and quoted original sources in their posts, more so than general bloggers from the Pew’s survey [6]. This was especially true for those participants who had published a scientific paper and were now successfully transferring conventions of scientific writing to their blogs. Additionally, it seems that reputation also played an important role, since those blogging under their real name followed these practices more than bloggers writing under a pseudonym.

Crucial differences, between our bloggers and those from other studies [6,16,19], were discovered regarding motivations for blogging. Sharing practical knowledge and skills, as well as influencing the way other people think, were major reasons for blogging among our medical bloggers, but not among general bloggers. A study of 177 general bloggers from Taiwan identified connecting with people and pouring out feelings to be major motivators for blogging [19]. To entertain the blogger and to allow the blogger to clarify thoughts and/or emotions were the most important reasons to blog for bloggers from the McKenzie study [16]. Staying in touch with family and friends was of great relevance to Pew bloggers [6] but not to those included in our study. Such dissimilarities could be, to a certain extent, explained by the very differences in main-blog topics. Medical bloggers responding to our survey predominantly wrote about topics aimed at their fellow colleagues, specialists in various health related fields, or patients. Only a small fraction of our bloggers constructed their blogs around their personal lives, which would be of far greater interest to their friends and families. On the other hand, those who had more than one blog identified communication with their friends and family as an important motivator. We could speculate that this is because, in blogs other than their main blog, they focus on topics aimed specifically at friends and family.

Our bloggers were extremely successful in drawing attention to their blogs. An astonishing 66% of them received attention from news media for their blogs, compared with mere 9% of Pew bloggers [6]. Responding medical bloggers offered their recipe for such success, which calls for more years of blogging, more hours spent on the Internet and getting medical news from other blogs. While persistence in blogging speaks for itself, getting medical news from other blogs is a great reminder of the importance of listening to what others have to say. It seems that news media find these popular stories originating from the blogosphere particularly interesting, perhaps because they frequently provide different and fresh perspectives.

### Identifying and Contacting Medical Bloggers

Identifying medical, or any other types of blogs, and determining their exact number is a challenging and hardly-achievable task. It is unknown how many medical blogs actually exist, and their number can only be estimated from the available data. According to Technorati [10], there were 5713 blogs labeled with the “medicine” tag on May 27, 2008. However, a quick check of some of these blogs reveals that medicine is not even a remote topic of most of them. We estimate that the actual number of active and English-language medical blogs is probably closer to 1000 to 2000. This inflation of supposed medical blogs on Technorati is in part influenced by the fact that bloggers are solely responsible for assigning up to 18 tags to their blogs, for better description and visibility. Such practice puts blogs with medicine as the main topic into the same category as those to whom authors have assigned such a tag after 17 other, more important, and appropriate ones.

In our research, we decided to utilize more reliable sources of medical blogs, which contrary to Technorati [10], require some form of verification by the editors and moderators prior to enlisting a blog submitted by its author. Following a thorough search of the Internet and reading of numerous posts on various medical blogs, we identified 4 such websites (Table 1). After we analyzed lists of blogs from these sources and removed duplicate entries, 627 unique medical blogs were identified. Surprisingly, 46% of these blogs were found to be either inactive, with latest posts on some being written even 1 to 2 years ago, or not to exist anymore. This fact clearly demonstrates the dynamic nature of the blogosphere, where huge numbers of new blogs emerge daily only to replace the ones abandoned by their once enthusiastic authors. The credibility of the identified websites listing medical blogs thus comes into question, since it is obvious that they are not fulfilling their primary role. We believe that, with only minor revisions, substantial improvements to these websites could be made to increase the reliability of their blog directories. Such measures should, among others, include regular visits to the listed blogs and removal of those inactive for a predetermined period of time. Proposed measures could easily be performed automatically, relieving the editors and moderators of such a repetitive and weary task. For the time being it seems that the best and most reliable sources of medical blogs are actually medical blogs themselves, as most of them have lists, so called blog rolls, of their favorite blogs. This was also confirmed in our study, since the most useful source among those we consulted, turned out to be a blog named Medgadget [20]. Only 2 out of 118 blogs nominated by other medical bloggers for the annual Medical Weblog Awards on Medgadget were demonstrated to be incorrect [21].

Almost all of the blogs we identified allowed their readers to post comments, but only 59% included the author’s e-mail address or an online contact form. Such a low percentage was somewhat unexpected, but it could be that bloggers felt the comment sections were sufficient enough for discussion and communication with their readers. Fear of spam messages is probably another reason why so many chose not to reveal their e-mail addresses. We noticed that a lot of bloggers who did provide them employed some sort of method, for example putting spaces in their e-mail addresses, intended to deceive spambots, which are automated computer programs collecting e-mail addresses from the Internet. Interestingly, bloggers who hosted blogs on their own personal websites more frequently provided direct contact information than others who used blog hosting services. These bloggers probably have considerable web programming skills and demand greater freedom and flexibility than the blog hosting services have to offer. Since the whole website is a product of their work and creativity, including contact information, if for nothing other than recognition, seems natural. Survey invitations were sent directly to selected bloggers, rather than being posted to comment sections of their latest blog posts, because we wanted only these bloggers to participate in the survey. It is easily conceivable that other medical bloggers not included in our sample could have, after reading such an invitation in comment sections of other blogs, decided to fill out the survey. This would completely disrupt the design of our study, as we would not know who our participants were, and whether their blogs met our inclusion criteria.

### Limitations and Future Studies

There were several limitations in our study, mainly concerned with our strategy of blog inclusion. At the time we conducted our research there were no comprehensive directories of medical blogs for us to use. Available websites listing medical blogs proved to have many drawbacks. Furthermore, a substantial number of bloggers were excluded from our study because direct contact information was not included in their blogs. Bloggers to whom we eventually sent survey invitations, replied in good numbers, but in lower numbers than we expected. We can speculate that such a response rate could be to some degree a consequence of overwhelming influx of data daily cramming their e-mail inboxes in which some interesting pieces of information can easily be overlooked. It would be useful for future research to conduct a follow up study to identify reasons why so many invited bloggers chose not to participate in the survey. We plan to ask bloggers to whom we originally sent invitations these questions when we notify them about the publication of our results.

During the course of the survey, many bloggers have contacted us to express their enthusiasm and compliment our efforts. Most importantly, all of them were very interested to see the results upon completion of the survey. Some even made it a condition of their participation in the survey that study results would be freely available. Bloggers are great supporters of open access to information, and this is one of the main reasons why we chose an open-access journal to publish our results. Hopefully their experience with our survey will make them more confident of similar research in the future.

It is clear that our results represent only bloggers who participated in the survey and may likely be skewed towards those who practice good blogging habits. Furthermore, we cannot be sure whether our respondents adequately represent all medical bloggers. Since this was a cross-sectional study, and given the fact that the blogosphere is highly dynamic, our results only present the situation at the time of the survey. Future studies should continue to evaluate medical bloggers and their blogs, presumably with larger numbers of participants included in prospective studies. These studies should also include blog-content analysis, as well as an investigation of the characteristics of blog readers. Additionally, survey questions should be further advanced. There should be more questions regarding the social, rather than technical, aspect of blogs, such as questions about the relationship with readers. Before such research can be successfully coordinated, the issue of medical blog directories, as depicted in our study, should be meticulously addressed. Recently, several new blog directories have emerged which have solved some of the issues mentioned in our study and may prove to be highly valuable for future studies [22,23]. Last, but not least, we are looking forward to see more research on medical blogs written in languages other than English to disclose their particularities and impact on local communities.

## Multimedia Appendix 1

37-item medical bloggers survey used in the study
