# Supplementary material for: Examining the Medical Blogosphere: An Online Survey of Medical Bloggers
Source: J Med Internet Res. 2008 Sep 23;10(3):e28. doi: 10.2196/jmir.1118 (PMC2626433; doi:10.2196/jmir.1118)
Supplement: Supplementary file 1 [file jmir_v10i3e28_app1.html]

PHPSurveyor


PHPSurveyor

|  |  |  |
| --- | --- | --- |
| **Data Entry** | | |
| **Medical Bloggers survey**  A survey to find out more about health/medical bloggers. To get a glimpse of their habits and opinions regarding their blogs. | | |
|  | **Date Stamp:** |  |
| **A) Internet habits** | | |
| Q1 | **Where do you go online most often?** | Work Home School Internet café Library Other public place Please choose.. Other Other: |
|  | | |
| Q2 | **What is your internet access speed?** | Dial-up  ISDN Cable Modem DSL T1/T3 Intranet Connection Do not know Please choose.. Other Other: |
|  | | |
| Q3 | **About how many hours per week do you spend on the Internet?** | Less then 1 1-5 5-10 10-20 20 or more Please choose.. |
|  | | |
| Q4 | **Do you read other medical/health blogs?** | Please choose.. Yes No |
|  | | |
| Q5 | **Are you subscribed to any medical/health podcasts?** | Yes No Do not know what podcasts are Please choose.. |
|  | | |
| Q6 | **Do you ever get medical/health news from?** | |  |  | | --- | --- | | the internet | Please choose.. Yes Uncertain No | | a blog | Please choose.. Yes Uncertain No | | email newsletters | Please choose.. Yes Uncertain No | | RSS feeds | Please choose.. Yes Uncertain No | | podcasts | Please choose.. Yes Uncertain No | | newspaper | Please choose.. Yes Uncertain No | | magazines | Please choose.. Yes Uncertain No | | TV | Please choose.. Yes Uncertain No | | radio | Please choose.. Yes Uncertain No | |
|  | | |
| Q7 | **What is your preferred source of medical/health news?** | Blogs Mass media Blogs and Mass Media Please choose.. |
|  | | |
| **B) Blog** | | |
| Q8 | **How many blogs do you have?** | One Two Three or more Please choose.. |
|  | | |
| Q9 | **Are you a single author of your primary blog?** | Please choose.. Yes No |
|  | | |
| Q10 | **Did you have a personal website before launching your blog?** | Please choose.. Yes No |
|  | | |
| Q11 | **For how long have you been blogging?** | Six months or less About a year Two or Three years Four or more years Please choose.. |
|  | | |
| Q12 | **In your opinion, will you still be blogging a year from now?** | Yes No I already stopped Not sure Please choose.. |
|  | | |
| Q13 | **Where do you usually blog from? (If other, please explain where)** | Home Work Other Please choose.. Other Other: |
|  | | |
| Q14 | **Do you usually blog on?** | Weekends Week days Weekends and Week days Please choose.. |
|  | | |
| Q15 | **How often do you post new material on your blog?** | Every day 3-5 days a week 1-2 days a week Every few weeks Every few months Please choose.. |
|  | | |
| Q16 | **How many hours per week do you spend updating your blog?** | Less then 1 1-5 5-10 10-20 20 or more Please choose.. |
|  | | |
| Q17 | **Do you blog about?** | One topic Several different topics Please choose.. |
|  | | |
| Q18 | **What would you say is the main topic of your blog?** |  |
|  | | |
| Q19 | **Do you blog under?** | Your real name Pseudonym or made-up name Please choose.. |
|  | | |
| Q20 | **Do you post?** | |  |  | | --- | --- | | photos | Please choose.. Yes Uncertain No | | images other then photos (clipart, graphs) | Please choose.. Yes Uncertain No | | audio files | Please choose.. Yes Uncertain No | | video files | Please choose.. Yes Uncertain No | |
|  | | |
| Q21 | **Do you allow your readers to post comments?** | Yes No Do not know Please choose.. |
|  | | |
| Q22 | **Do you offer a feed (RSS) service to your readers?** | Yes No Do not know Please choose.. |
|  | | |
| Q23 | **Do you offer a mailing list subscription to your readers (email newsletter)?** | Yes No Do not know Please choose.. |
|  | | |
| Q24 | **From whom have you received attention for your blog? (choose all that apply)** | Other bloggers |
|  | | |
| Q25 | **Have you ever experienced any problems at work/school because of your blog? (if yes, please state them)** | Yes No Please choose..  Comment: |
|  | | |
| Q26 | **Do you keep traffic statistics for your blog? (if yes, how many hits do you receive per day)** | Yes No Do not know Please choose..  Comment: |
|  | | |
| **C) Writing** | | |
| Q27 | **Please tell us is this is the reason you personally blog, or not:** | |  |  | | --- | --- | | To stay in touch with friends and family | Please choose.. Major reason Minor reason Not a reason | | To network or meet new people | Please choose.. Major reason Minor reason Not a reason | | To make money | Please choose.. Major reason Minor reason Not a reason | | To improve your writing skills | Please choose.. Major reason Minor reason Not a reason | | To store resources of information that is important to you | Please choose.. Major reason Minor reason Not a reason | | To motivate other people to action | Please choose.. Major reason Minor reason Not a reason | | To express yourself creatively | Please choose.. Major reason Minor reason Not a reason | | To document your personal experiences or share them with others | Please choose.. Major reason Minor reason Not a reason | | To share practical knowledge or skills with others | Please choose.. Major reason Minor reason Not a reason | | To influence the way other people think | Please choose.. Major reason Minor reason Not a reason | | To entertain people | Please choose.. Major reason Minor reason Not a reason | |
|  | | |
| Q28 | **Please tell us how often, if ever do you do the following things on your blog?** | |  |  | | --- | --- | | Get permission to post copyrighted material | Please choose.. Often Sometimes Hardly ever Never Does not apply to me Do not know | | Quote other people/media directly | Please choose.. Often Sometimes Hardly ever Never Does not apply to me Do not know | | Spend extra time trying to verify facts | Please choose.. Often Sometimes Hardly ever Never Does not apply to me Do not know | | Post corrections | Please choose.. Often Sometimes Hardly ever Never Does not apply to me Do not know | | Include links to original source of material | Please choose.. Often Sometimes Hardly ever Never Does not apply to me Do not know | |
|  | | |
| Q29 | **Have you ever published?** | |  |  | | --- | --- | | Book or a chapter in a book | Please choose.. Yes Uncertain No | | Newspaper article | Please choose.. Yes Uncertain No | | Scientific paper | Please choose.. Yes Uncertain No | |
|  | | |
| **D) Personal data** | | |
| Q30 | **Are you male or female?** | Please choose.. Female Male |
|  | | |
| Q31 | **How old are you? (in years)** | <17 18-29 30-49 50-64 65> Please choose.. |
|  | | |
| Q32 | **Your race/ethnicity** | American Indian and Alaska Native Asian Black Native Hawaiian and other Pacific Islander White Please choose.. Other Other: |
|  | | |
| Q33 | **What is the last year of formal education you completed?** | Some high school or less Graduated high school Vocational or technical school Some college Graduated college Some postgraduate college Master's degree or doctorate Please choose.. |
|  | | |
| Q34 | **What is your job title? e.g. Physician, Paramedic, Medical student** |  |
|  | | |
| Q35 | **Industry you are in? e.g. Healthcare, Publishing, Computer....** |  |
|  | | |
| Q36 | **What is your specialty? e.g. Cardiology, Family medicine, Pediatrics......** |  |
|  | | |
| Q37 | **In which country are you currently residing?** |  |
|  | | |
|  | | |
|  |

 
